# Identifying dramatic selection shifts in phylogenetic trees

**DOI:** 10.1186/1471-2148-7-S1-S10

**Published:** 2007-02-08

**Authors:** Karin S Dorman

**Affiliations:** 1 Departments of Statistics and Genetics Development & Cell Biology, and the Program in Bioinformatics and Computational Biology, Iowa State University, Ames, IA, USA

## Abstract

**Background:**

The rate of evolution varies spatially along genomes and temporally in time. The presence of evolutionary rate variation is an informative signal that often marks functional regions of genomes and historical selection events. There exist many tests for temporal rate variation, or heterotachy, that start by partitioning sampled sequences into two or more groups and testing rate homogeneity among the groups. I develop a Bayesian method to infer phylogenetic trees with a divergence point, or dramatic temporal shifts in selection pressure that affect many nucleotide sites simultaneously, located at an unknown position in the tree.

**Results:**

Simulation demonstrates that the method is most able to detect divergence points when rate variation and the number of affected sites is high, but not beyond biologically relevant values. The method is applied to two viral data sets. A divergence point is identified separating the B and C subtypes, two genetically distinct variants of HIV that have spread into different human populations with the AIDS epidemic. In contrast, no strong signal of temporal rate variation is found in a sample of F and H genotypes, two genetic variants of HBV that have likely evolved with humans during their immigration and expansion into the Americas.

**Conclusion:**

Temporal shifts in evolutionary rate of sufficient magnitude are detectable in the history of sampled sequences. The ability to detect such divergence points without the need to specify a prior hypothesis about the location or timing of the divergence point should help scientists identify historically important selection events and decipher mechanisms of evolution.

## Background

The rate of evolution at a site at one moment in time depends on the underlying mutation rate and the overlying selective constraints. Both determinants of evolutionary rate may change spatially, along the genome, or temporally, in evolutionary time, to produce evolutionary rate variation. Many highly important biological processes manifest in sequence data as spatial or temporal rate variation, and this signal is often harnessed to extract biological information from sampled sequences. Sites in a gene with high nonsynonymous rates may be responding to positive selection (e.g. [1-3]), while conserved genomic sites likely carry out mission-critical biological functions (e.g. [4,5]). Temporal shifts in evolutionary rate suggest functional change and may be used to explain the evolution of novel traits (e.g. [6,7]), to characterize functional innovation within gene families (e.g. [8-11]), and to resolve phylogenetic discrepancies (e.g. [12,13]).

As soon as molecular sequence data were available, evolutionary rates were observed to vary among sites. Some amino acid positions seem completely invariant in proteins [14], and a nucleotide model with an unknown fraction of invariant sites better approximates mitochondrial data [15]. This two-class, variable or invariant, site classification can generalize to any discrete distribution of rates across site [16]. Theory and simulations suggest that phylogenetic inference is particularly sensitive to unrecognized site-to-site rate variation [17,18]. Models that incorporate spatial rate variation often fit biological sequence data statistically better than models that assume a constant rate [19-24]. Probably the most common model for site-to-site rate variation is the discrete approximation to gamma distributed rates [25], but all these models are collectively referred to as RAS models for rates across sites.

Temporal rate variation, also known as heterotachy, is not so easy to observe directly, but it is a long-standing idea [26,27]. Early work produced the word covarion, *co*ncomitantly *var*iable codo*n*, also extended to nucleotides [28], to name the concept of temporal rate variation. In the original covarion model an approximately constant fraction of sites evolve, accumulating variation, but the site membership in the variable pool is continuously changing in time. The covarion model experienced very little theoretical progress until formalized as a Markov model in 1998 [29]. Then, phylogenetic implementations followed rapidly [30,31]. Recently, the covarion model was extended to allow switching between more than two site classes [32]. Heterotachy models have largely assumed the switching rate is constant throughout time.

There is increasing evidence that heterotachy is an important evolutionary phenomenon, perhaps exceeding or superseding the importance of site-to-site rate variation [33,34]. Much of this evidence is generated by testing the hypothesis of equal rates between pre-defined clades, for which many tests have been derived [10,11,31,33,35-40]. An alternative strategy attempts to detect brief spurts of evolution presumed to occur coincident with functional innovation. Such episodic evolution leaves signatures on branches of phylogenetic trees, where the ratio of nonsynonymous to synonymous substitution rates exceeds one [41-43]. Application of both kinds of techniques to large data sets reveals widespread heterotachy [44,45]. These tests are undoubtedly most sensitive to dramatic shifts in selection pressure, where many sites simultaneously experience an altered evolutionary rate. Gene duplication, environmental changes, and niche invasion are all associated with large-scale changes in selection pressure affecting many sites in a genome. There are two questions of interest: (1) whether selection shifts occur, for example after gene duplication, and (2) when selection shifts occur in the history of sequences, e.g. to time historical niche invasion based on a sample of extant species. Henceforth, we shall call the locations of these shifts *divergence points *in time or phylogenetic trees.

Gu [36] describes a maximum likelihood method for detecting divergence points associated with gene duplication. Given a set of homologous genes categorized into paralogous groups separated by gene duplication and the phylogenetic trees that relate orthologs within groups, the method can identify amino acid positions that are functionally divergent between the groups. It works by hypothesizing that a fraction *θ *of amino acid sites (*divergent sites*) acquire independent function and thereby *independent *evolutionary rates in two or more of the ortholog groups. The remaining *constrained *sites retain function and evolve at the same *dependent *rate in all groups. After maximum likelihood estimation of *θ*, subtree branch lengths, and evolutionary parameters, Bayes rule can predict functionally important residues if the estimate  is significantly bigger than zero.

The present paper extends the Gu method by performing inference on a full tree without specifying *a priori *the branch where a divergence point is expected. I develop the method in a Bayesian context for nucleotide sequences and test it using a panel of simulated sequences. I then apply the method to study divergence between Human Immunodeficiency Virus (HIV) subtypes and Hepatitis B Virus (HBV) genotypes, revealing a substantial selection event sometime after separation of HIV subtypes B and C and no evidence of a selection event separating HBV genotypes F and H.

## Results and Discussion

### Simulation

#### Varying the magnitude of the selection shift

**Figure 1:**
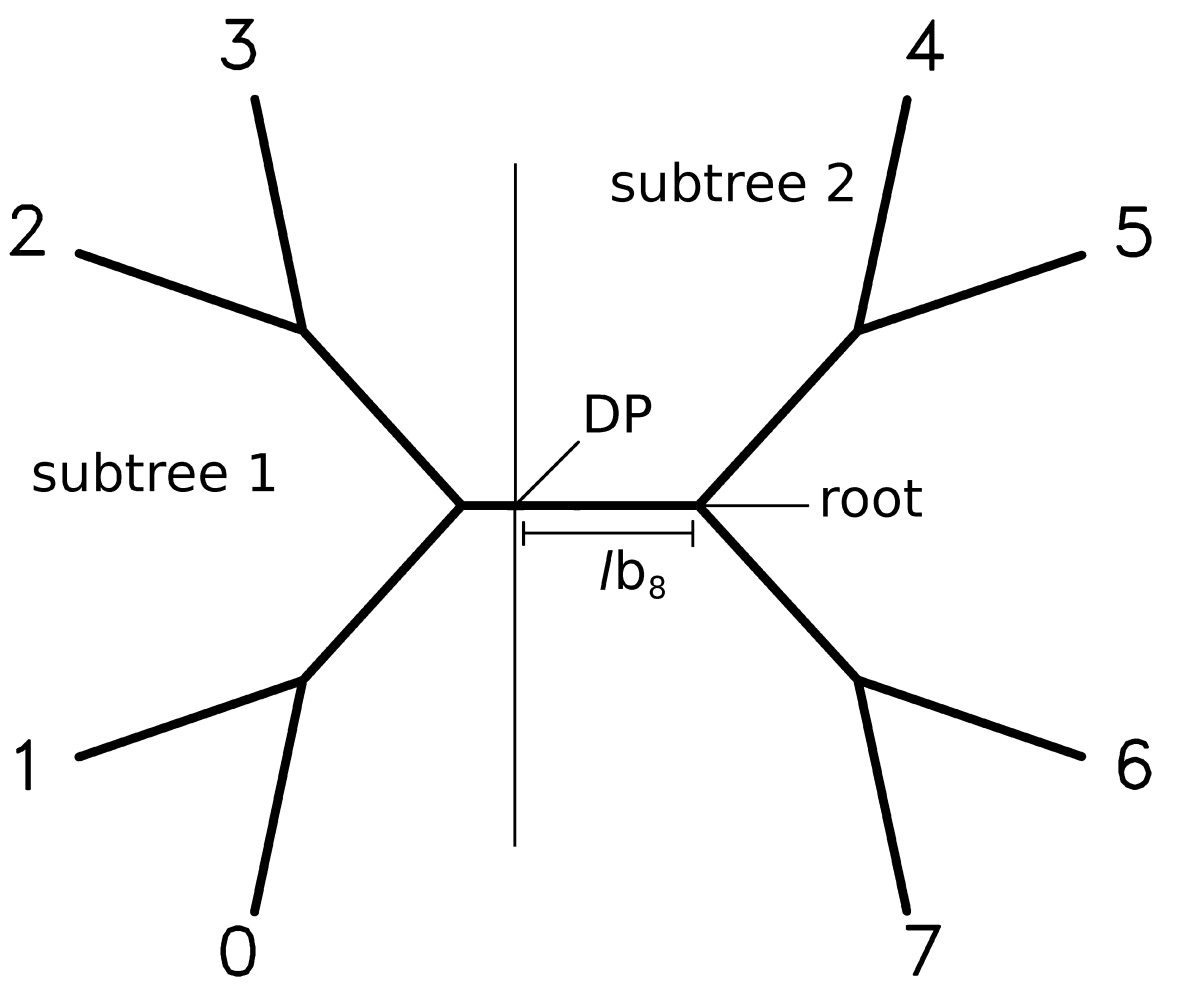
**Simulation tree**. The phylogenetic tree used for simulation. There are eight taxa, labeled 0 to 7, related according to the depicted topology with all branch lengths equal. Each simulation assumes a single divergence point (DP) located at a distance *l *= 0.9 away from the right end of the middle branch, known as branch 8. The vertical line, splits the full phylogeny into two subtrees. Subtree 2 has a stubbed branch where it connected to subtree 1 of length *lt*_8_. Subtree 1 has a stubbed branch of length (1 – *l*)*t*_8_.

I simulate data sets assuming a single divergence point in a topology relating eight hypothetical taxa (Figure 1). I use a discrete gamma distribution with four rate categories and the same shape parameter *α *to approximate the variation in both spatial and temporal rates [25]. To explore the sensitivity of the method to the strength of the temporal selection shift, I vary the degree of rate variation (smaller *α *implies greater rate variation) and the fraction of sites *θ *experiencing a selection shift across the divergence point. When a site is selected to shift rate, it randomly selects a new rate class from the discrete gamma distribution. All other parameters do not vary in this first set of simulations. In particular, the simulated divergence point is located at relative position *l *= 0.9 on branch *b *= 8 in the topology *τ *of Figure 1 with *t*_*j *_= 0.1 expected mutations (transition/transversion ratio *κ *= 2) per site along each branch *j *= 1,...,13. C code implementing Markov chain Monte Carlo sampling of the posterior distribution analyzes each simulated alignment. Posterior statistics of model parameters are computed along with the Bayes factor *B*_*DP *_in favor of a divergence point somewhere in the tree. When log_10_*B*_*DP *_> 1, there is strong support for a divergence point, which then allows conditional estimation of *θ*, *l*, and the Bayes factor *B*_*j *_favoring a divergence point located specifically on branch *j*. All these latter statistics are based on the subset of MCMC samples that have a divergence point.

**Figure 2:**
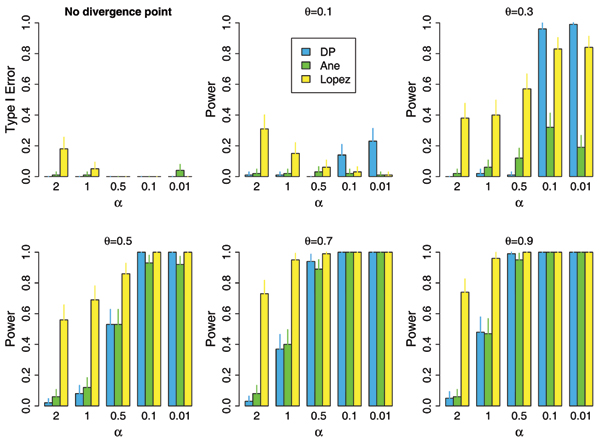
**Method comparison**. The type I error rate and power of the new method are compared with two other methods, that of Ané et al. [33] and Lopez et al. [13]. The null hypothesis is no temporal rate variation or homotachy. Type I error means the method rejects the null when it is actually true. Power is the probability the method correctly rejects the null when there actually is a divergence point. For the Bayesian method, the null hypothesis is rejected if log_10 _*B*_*DP *_> 1. Error bars indicate 95% confidence intervals accounting for simulation error.

Figure 2 plots the method type I error rate and power for the various simulation conditions (blue bars) when the null hypothesis is homotachy. Here, type I errors result when there is no divergence point, but the user concludes one because log_10 _*B*_*DP *_> 1. Type I errors do not occur for any of the 500 simulations without a divergence point. Power is the probability that the method strongly supports a divergence point when one is simulated. For frequentist methods, the type II error rate is one minus the power, i.e. the probability of accepting the null hypothesis when the alternative hypothesis of heterotachy is actually true. Bayesian analyses are advantageous when it comes to assessing the strength of the null hypothesis. In this case, one should not commit to the null hypothesis of homotachy unless it receives strong support, e.g. log_10_*B*_*DP *_< -1, which here occurs for only nine of 2500 datasets simulated with a divergence point and only when *θ *= 0.1. A more important concern for the Bayesian method is the decreasing power to detect the divergence point as rate variation and the fraction of sites subject to rate shifts decrease. When *θ *= 0.1, the divergence point becomes effectively undetectable. For all other simulated values of *θ*, the divergence point is detectable given sufficient rate variation. When *α *= 2, the method never works well, and the rates for the four discrete categories are 0.3, 0.7, 1.1 and 2.0, yielding less than seven-fold differences in rate. Susko et al. [11] use a regression technique to estimate the size of rate differences between eukaryotic and archaebacterial amino acid sequences of elongation factor 1*α *and find rate variation roughly between 3 and 15-fold, just straddling the level of rate variation detectable in this simulation.

When a simulated divergence point is highly supported, the identification of the branch with the divergence point is exceptionally successful via Bayes factor *B*_*j *_for branch *j*. Out of 1195 simulations with high support for the divergence point, only 4 failed to also identify the true branch 8 as highly likely (log_10 _*B*_8 _> 1) to carry that divergence point. Only twice, another branch is incorrectly found to strongly favor a divergence point somewhere along its length. These results demonstrate the method can not only detect the presence of a divergence point in a phylogenetic tree, but also pinpoint the affected branch with high confidence.

**Figure 3:**
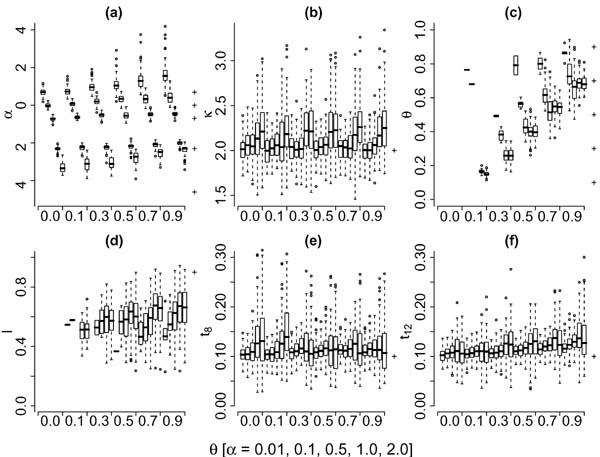
**Parameter estimation**. Boxplots of posterior mean estimates of (a) *α*, (b) *κ *(c) *θ*, (d) *l*, (e) branch length of the 8th branch *t*_8 _(the one with the divergence point), and (f) branch length of the 12th branch *t*_12_. Each boxplot is based on 100 simulations, except (c) and (d), where posterior means of *θ *and *l *are only estimated for those simulations strongly supporting the divergence point. Results are grouped by simulated *θ *value as marked on the x-axis. There are five simulations per group, with simulated *α decreasing *2.0, 1.0, 0.5, 0.1, 0.01. The arrangement is such that temporal rate variation is generally increasing from left to right. The estimates of *α *are logged before plotting to better show the variation in the smaller values. The location of the true value(s) of each parameter are marked by a + just right of its plot.

Table 1 records the posterior mean (indicating accuracy) and width of the 95% Bayesian credible intervals (indicating precision) for parameters *α*, *θ *and *l *averaged across simulated data sets. Figure 3 plots the distributions of posterior means for these parameters as well as *κ *and two branch lengths: *t*_8 _is the length of the branch carrying the divergence point and *t*_12 _is that of a randomly selected terminal branch. Each entry in Table 1 and boxplot in Figure 3 is based on 100 estimated values except those for *θ *and *l*, which may be estimated in far fewer simulations. Estimates of *α*, which are logged before plotting in Figure 3(a), tend to overestimate the true value, especially when true *α *= 0.01 and as the fraction of heterotachous sites increases. Evolutionary rate parameter *κ*, the transition/transversion ratio, is fairly well estimated but with a slight upward bias when site-to-site rate variation is high (*α *< 0.5). In contrast, estimation of *θ *is poor. While there is relatively low posterior uncertainty in *θ *(as compared to *l*), the estimates are dramatically and increasingly downward biased as true *θ *climbs above 0.1. The effect is not just a consequence of the prior, which would tend to pull estimates toward the prior mean of 0.5, because even for true *θ *≤ 0.5, the bias is downward. The bias is most noticeable for those datasets that detect the divergence point. When simulating *θ *= 0.9 and *α *= 0.01, the divergence point is always detected with high confidence, but the 95% Bayesian credible intervals for *θ *never contain the true value. For estimating the divergence point location *l*, the fact that the estimates pull toward the true value 0.9 as heterotachy increases suggests that there is some information in the data about this parameter, however the information is weak as demonstrated by the very wide Bayesian credible intervals in Table 1. Given this result, a model that simply places divergence points at internal nodes of the tree may have just as much power to detect divergence events, while simplifying the MCMC algorithm and convergence. Finally, estimates of all branch lengths tend to be less precise with increasing site-to-site rate variation. In addition, branch 12 is increasingly overestimated as the amount of noticeable heterotachy increases. Since temporal and spatial rate variation can be somewhat or completely confounded [29], it is not surprising to find estimation of *α *and *θ *somewhat entangled. Additionally, failure to adequately account for site-to-site rate variation, in this case because *α *is overestimated, is known to produce biased branch length estimates [46].

Whether the performance in simulation translates to real biological sequences is still questionable. Previous analyses suggest that biological site-to-site rate variation falls in the range *α *∈ (0.1, 10), with nonsynonymous (and more likely *selected*) rate variation tending to fall below *α *= 1 [24,46,47]. While there is less information about biologically relevant ranges for *θ*, Gu [36] estimates *θ *= 0.46 for a study of the cyclooxygenase gene family at the amino acid level. Comparison of rates between pre-defined monophyletic groups shows very high proportions of sites eventually experience heterotachy during evolution, even in functionally conserved sequences, for example, 66% of rRNA sites [48] or as high as 47% of the cytochrome *b *amino acids [49]. Yang and Nielsen [42] find the proportion of codons undergoing positive selection during episodic evolution along particular lineages to be between 0.03 and 0.2, depending on the gene analyzed. Thus, it appears that the power of this method to detect divergence points may falter near the boundary of biological relevance. In the next section, additional simulations investigate the amount of information, as measured by the sequence divergence, alignment length, and number of taxa, needed to detect divergence points.

#### Varying the amount of data and evolution

**Figure 4:**
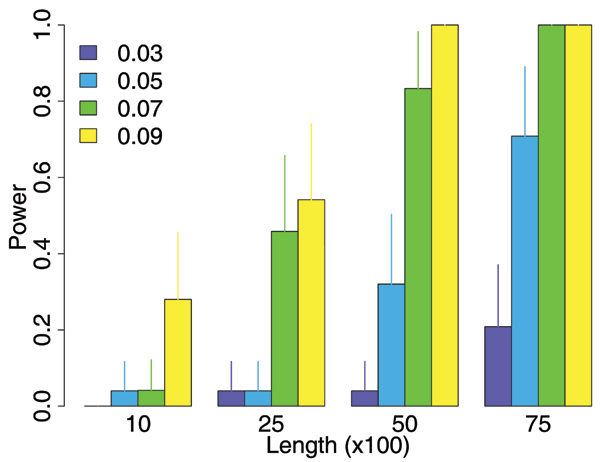
**Sample size and power**. The proportion of simulations that strongly support the simulated divergence point when *α *= 0.7 and *θ *= 0.5. Each group of bars corresponds to a different alignment length ranging through 1000, 2500, 5000, and 7500. Within the group, there are four different branch lengths assigned to every branch in the simulation topology of Figure 1, either 0.03, 0.05, 0.07, or 0.09. Error bars indicate 95% confidence intervals accounting for simulation error.

To explore the power of the method to detect the divergence point for varying amounts and diversity of input data, I generate simulated data sets under a variety of conditions. I start by again simulating data alignments using the tree of Figure 1. For these simulations, I set *α *= 0.7 and *θ *= 0.5 and vary both the branch length (all branches of the topology are equal) and the length of the alignment. Figure 4 displays the results, showing that increasing diversity, as measured by the branch length, and data, as measured by the alignment length, both improve the power of the method to detect the divergence point. In particular, the divergence point is detectable for these *α *and *θ *when the branch length is above 0.07 and the alignment length is over 5000. Similar patterns are observed for different *α *and *θ *combinations. As long as *θ *≥ 0.3, the method achieves good power at least for the simulation with *t*_*j *_= 0.9 and 7500 base pairs (data not shown).

**Figure 5:**
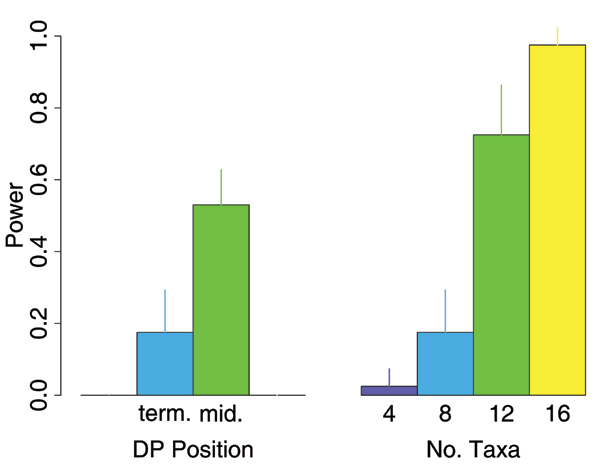
**Power as a function of group size**. The power of the method to detect the divergence point with strong support as the branch location of the divergence point or the size of the topology changes. For the first set, the divergence point is located on the middle branch or a terminal branch of the 8-taxa tree of Figure 1 and *α *= 0.5, *θ *= 0.5, *t*_*j*_*= *0.1, and *L = *1000. For the second set, the divergence point is located on the middle branch of a 4-, 8-, 12-, or 16-taxa tree, and *α *= 0.1, *θ *= 0.1, *t*_*j *_= 0.1, and *L *= 1000. Error bars indicate 95% confidence intervals accounting for simulation error.

To test the impact of including more sequences, I simulate data with increasing numbers of taxa in each subtree. This time, *α *= 0.5, *θ *= 0.5, and all branch lengths *t*_*j *_= 0.1 are selected to demonstrate a range of outcomes in resulting power. The original simulation tree of Figure 1 has 8 taxa. I also simulate sequences with 4, 12, or 16 taxa, maintaining the divergence point on the middle branch and adding taxa in a balanced fashion to both subtrees. Because all branch lengths are held constant, any effect of adding more taxa could be a consequence of the additional taxa or the increase in total evolutionary time simulated. The power of the method to detect the divergence point increases substantially with the number of taxa in each subtree (Figure 5). Unfortunately, the computational cost also increases substantially. Roughly, based on informal observation only, 8 taxa take ten times as long as 4 taxa, and computational times double for every 4 additional taxa after that. Finally, I also examine the probability of detecting heterotachy when the divergence point is placed on a terminal branch rather than the internal branch of Figure 1. This time *α *= *θ *= *t*_*j *_= 0.1. Not surprisingly, power of the method to detect a terminal branch divergence point is substantially compromised (Figure 5), indicating that balanced subtrees including many taxa provide the ideal conditions for detecting a divergence point.

#### Comparison to existing heterotachy detection methods

Figure 2 compares the power of the Bayesian divergence point method with two other statistical tests for heterotachy given pre-defined subgroups. Ané et al. [33] recently describe a parametric bootstrap test of the covarion model that tests the degree of independence in the proportion of invariant sites in the two subgroups. When applied to the first set of simulated data, this method demonstrates a low type I error rate in the absence of heterotachy and comparable power to the Bayesian method in the presence of heterotachy except when *θ *= 0.1 and *α *is small. However, the method is not ideally matched to the simulations since it specifically tests the covarion model with invariant sites, but the simulation model allows no truly invariant sites. A more appropriate test is suggested by Lopez et al. [49], who describe a method to compare the number of substitutions in each subgroup at each site. Under the homotachous model, the number of substitutions at a site should be proportional to the amount of evolution, or tree length, of each subtree. Substantial deviations from this expectation, as measured by a chi-square statistic, indicate a change in evolutionary rate between the two subtrees. As expected, this method has more power than the Ané et al. and also beats the Bayesian method. In particular, it is better able to detect heterotachy when *α *> 0.5 and there is low site-to-site rate variation. However, these conditions are also the ones where the method's type I error rate begins to exceed expectation (see Figure 2, No divergence point and *θ *= 0.1). Thus, it may be that the conservative behavior of both the Ané et al. and Bayesian methods in the presence of low rate variation are desirable.

### HIV

As HIV spread into the human population in the last century, genetically distinct lineages arose [50]. These so-called subtypes have distinct geographic distributions [51]. In particular, subtype B dominates throughout much of the non-African and non-Asian world, while subtype C dominates in southern and eastern Africa, parts of the Middle East, and India [51]. Much of the geographic restriction of subtypes can be explained by the travels of a few infected individuals [52], however there is also evidence of population level selection on the virus, particularly in relation to immune selection [53]. I hypothesize that if the virus encounters substantial population-specific selection pressures when entering a new population, a selection shift signature may be detectable on the branches of phylogenetic trees that separate subtypes.

**Figure 6:**
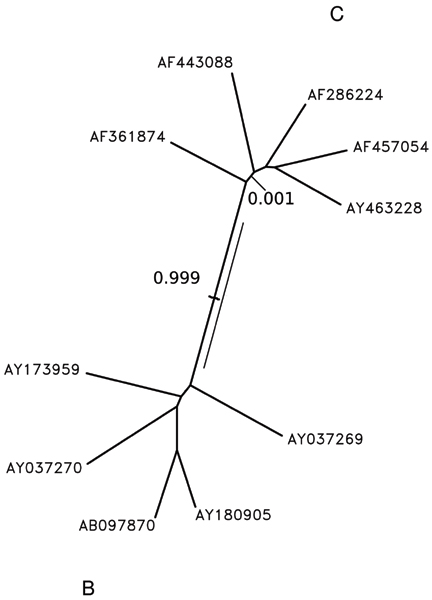
**Phylogenetic tree of HIV subtypes B and C**. Phylogenetic tree inferred from HIV data. The topology is not estimated, but branch lengths are shown at their posterior means. The posterior mean location of the divergence point is shown along with a parallel bar demarcating the 95% Bayesian credible interval. The numbers indicate the conditional posterior probability that the indicated branch carries the divergence point given there is a divergence point in the tree. For reference, the length of the middle branch is 0.12.

To test the hypothesis, I align 10 HIV sequences, five from subtype B and five from subtype C. Summaries of the marginal posterior distributions for each continuous parameter of the model are shown in Table 2. The reported potential scale reduction factors [54] demonstrate healthy agreement between the six independent MCMC runs and all six runs are combined for statistical estimation. The Bayes factor in favor of a divergence point cannot be computed because the support for a divergence point is unanimous in the posterior sample. The model clearly identifies a highly supported divergence point on the branch separating subtypes B and C, with log_10 _*B*_*BC *_= 4.08, where indexing is meant to indicate the branch separating B and C. Figure 6 shows the location of the estimated divergence point along with its 95% Bayesian credible interval on the phylogeny drawn with branch lengths at their posterior means. The precise location of the divergence point along the branch is poorly estimated, but the selected branch is highly supported. Considering the estimated values of *α *= 0.23 and *θ *= 0.28, the alignment length *L *= 6610, and the average branch length ( = 0.04) of this data set, simulation results (not shown) suggest that the method *just *has enough power to detect the presence of a divergence point. It may not be possible to detect heterotachy for shorter regions of HIV.

### HBV

Like HIV, HBV has diverged into genetically distinct lineages with nonuniform geographic distribution around the world [55]. In the case of HBV, these lineages are called genotypes. Although the origins of HBV are unclear, HBV is most likely to have evolved with humans since our emigration from Africa [56]. The genotypes and their geographic distribution can thus be associated with major migration events, but it remains unclear whether the genotypes express distinct disease phenotypes [57-59]. HBV genotypes F and H are restricted to the Americas, probably arriving on these continents with the first human immigrants [57]. Genotype H is found much less frequently than F, and its origin is uncertain [60]. In fact, its classification as a separate genotype is controversial [61]. Given the best estimate of HBV origins, it is not likely that the spread of HBV into new human populations has exerted recent selective pressure on the virus, however co-evolution of the virus along with the human host may create divergence points along branches where humans and viruses co-adapted to new ecological niches.

**Figure 7:**
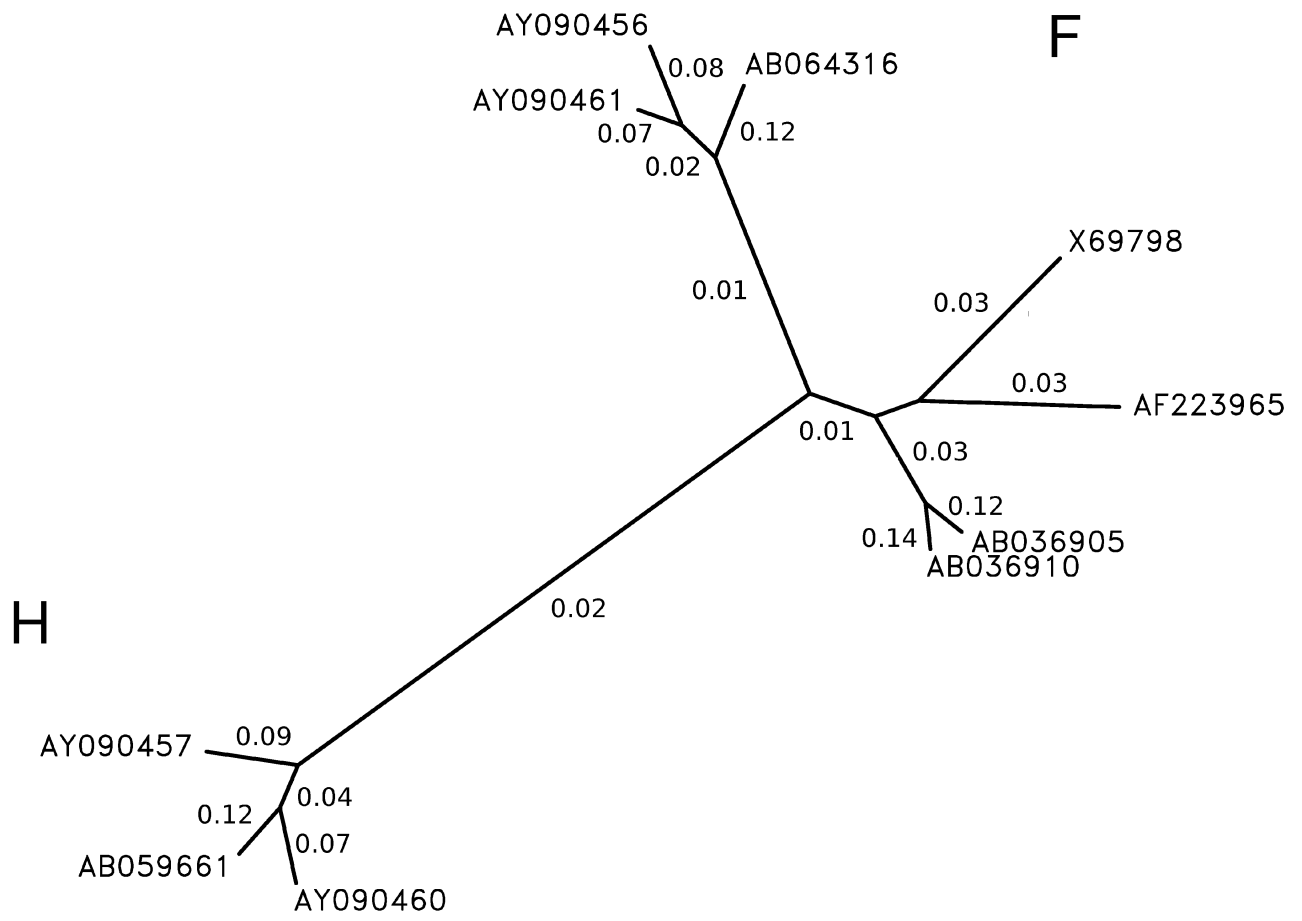
**Phylogenetic tree of HBV subtypes F and H**. Phylogenetic tree inferred from HBV data. The topology is not estimated, but branch lengths are shown at their posterior means. The numbers by each branch indicate the conditional posterior probability that the indicated branch carries the divergence point given a divergence point is present in the tree. In fact, there is substantial support against a divergence point in this data set. For reference, the length of the middle branch is 0.07

To look for divergence points related to the emergence of HBV genotypes F and H, I align seven genotype F sequences and three genotype H sequences. Posterior summaries are in Table 3. Figure 7 displays the estimated phylogeny relating these 10 sequences with the branch lengths drawn proportional to their posterior means. The number accompanying each branch is the conditional posterior probability that the divergence point lies somewhere along that branch given one exists somewhere in the tree. In contrast to the HIV results, a divergence point is not supported by the data with log_10 _*B*_*DP *_= -0.64. Considering only the posterior sample supporting a divergence point (1123 samples), no branch shows evidence of strong heterotachy, although the posterior distribution across branches is significantly different from the uniform prior (p-value < 0.001). The alternative hypothesis of homotachy is substantially, but not strongly supported, and the method may simply have insufficient power to detect heterotachy in this data set. Notably, because H is a poorly sampled genotype, the three representatives included here are highly similar, thereby forcing any potential genotype-associated divergence point onto what is effectively a terminal branch. Table 4 suggests power is low under this condition, but I performed no simulations with parameters matching the HBV data, so it is unclear whether the method should have sufficient power to estimate the presence of a divergence point. Evidence of site-to-site rate variation is high, with the posterior mean *α *= 0.04, however the low diversity (average branch length 0.015) and short alignment (3,215 base pairs) sharply reduce the power of the method.

In addition, strong spatial rate variation may not translate to strong temporal rate variation in the case of HBV. Normally, the magnitude of temporal rate variation is expected to approximately match the magnitude of spatial rate variation, because choosing a new function for a site is roughly equivalent to selecting a new site at random from the same protein [29,62]. The model makes this assumption by using the same rate class distribution for spatial and temporal rate variation. Strong purifying selection combined with an error-prone reverse transcriptase is expected to produce highly heterogeneous rates in HBV, with widespread conservation due to overlapping reading frames interrupted by a limited number of mutation-tolerant sites [63]. But for a dual-coding nucleotide to temporally shift rate class, it must acquire a new function in both reading frames. This dual constraint may eliminate the possibility of divergence points in HBV and certainly reduces both the magnitude of temporal rate shifts and the number of affected sites. In short, the biology of HBV may limit both the presence of and the power to detect divergence points. Increasing the number of sampled sequences per genotype may restore power, but this option is not examined further here.

## Conclusion

Spatial and temporal rate variation is the signature left on genomic sequences by the force of selection. Although mechanistic differences can also generate evolutionary rate variation, it appears that the selection signal is stronger [24]. A flurry of new methods have emerged to detect and utilize this signal to inform on function [43,64,65]. I propose a new method for inferring the location of divergence points in phylogenies. The method joins a host of existing tests to detect selection sweeps along pre-specified branches of a phylogeny [10,11,31,33,35-40,42,43]. Unlike existing methods, however, the proposed method does not require *a priori *branch specification.

The method is developed in a Bayesian context and applied to two viral data sets. The HIV data strongly support the presence of at least one divergence point, while the HBV data fit a model with substantial site-to-site rate variation but no sudden or dramatic temporal rate shifts. Heterotachy may still occur in the HBV sequences, perhaps more subtly, accumulating slowly and methodically over time as in the covarion model [29]. The presence of a divergence point between HIV subtypes B and C indicates a profound rate change *after *the split of these two subtypes. It does not prove that the divergence point occurred concurrent with the split or caused it in any way. However, it is plausible that host population differences are related to the apparent selection differences, and further testing may reveal specific cause-and-effect relationships between host differences and rate differences in the HIV genome. Finally, I make no effort to detect the possible presence of other, weaker divergence points in this set of HIV data. A natural extension of the current Bayesian method is to allow and estimate more than one divergence point per phylogenetic tree. Interestingly, the episodic evolutionary events sought by methods that detect high nonsynonymous rates on particular branches [42,43] consist of *two *divergence points appearing close to or on the same branch of a phylogeny. Thus, multi-divergence point models may be better equipped to detect and quantitate temporary shifts in evolution. In the limit as the number of divergence points *d *increases and the proportion of diverging sites *θ *decreases, the model approaches the covarion model.

Recombination between subtypes/genotypes is a common phenomena in both HIV [66] and HBV [67] that can result in a non-constant topology or branch lengths along an alignment. Another oversight that could lead to non-constant branch lengths along the alignment is the fact that both data sets consist of multiple genes. While site-to-site rate variation can account for some rate variation along the alignment, it does not adequately model whole-gene shifts in rate that can result when different genes evolve according to different processes. In assuming that a fixed topology and a single set of branch lengths applies to the full alignment, I prohibit the possibility of recombination or gene effects and may force the wrong topology and or branch lengths on some sites if these assumptions are not met. Forcing either an incorrect topology or incorrect branch lengths could affect inference of heterotachy. The first set of simulations and Figure 2 demonstrate that branch length estimation can depend on the estimation of heterotachy and rate variation. The reverse must also be true, such that incorrect branch lengths and especially topology, could influence inference on *θ*, *l*, and possibly even the presence of a divergence point. To limit the possibility of such an artifact, all selected viral sequences had been previously reported as nonrecombinant or verified so using recombination detection software [68]. This step insures that the two subtrees are consistent throughout the alignment, however it does not guarantee that the subtree topologies are consistent. Another natural extension of the current model is to include recombination models that allow topology and branch length variation along the alignment while simultaneously estimating heterotachy. Both models are already computationally difficult when considered separately, and combining them obviously represents a major computational challenge.

The current approach does not estimate the phylogenetic tree, assuming that it can be derived confidently using other methods. This assumption requires serious reconsideration since heterotachy can affect phylogenetic tree estimation [13,69]. In addition, including additional taxa should improve the power of the method to detect selection and estimate *θ*, yet adding taxa increases the chance of topological uncertainty. The ideal solution is to simultaneously estimate phylogeny and divergence point location. Unfortunately, because branches do not retain definition across different topologies, divergence points may also lose definition. Presumably, however, a simultaneous estimation procedure should be able to detect strong divergence points on supported branches while allowing for topological uncertainty within clades.

A persistent question lurking behind these analyses is whether detected rate variation is actually connected to selection and function [70]. Lopez et al. [49] suggest that temporal rate variation need not relate to function because even mitochondrial cytochrome *b*, whose function is highly conserved among all vertebrates, tests positive for heterotachy. Comparison of vertebrate *α *and *β *globins revealed a similar disconnect between significantly heterotachous sites and those sites most likely responsible for protein functional differences [71], leading Philippe et al. [72] to conclude heterotachy may be a largely neutral evolutionary process on alternative, but viable protein conformations. In order to identify functionally important rate shifts, it may be necessary to design models that separate this neutral heterotachy, e.g. covarion-like models, from the sudden and temporary heterotachy of the type expected after gene duplication or other environmental shifts. Yet even these models may be over-simpistic. Protein models [73,74] that allow amino acid sites to self-classify into highly flexible evolutionary classes, reveal that sites with different functional or structural jobs differ not only in their evolutionary rates, but also in how they mutate. The divergence point described here allows no such changes in site properties. For example, if transitions are much more likely than transversions in one rate class, they are identically skewed in all other rate classes. Yet it is plausible that the skew will shrink at certain codon positions or within some amino acid contexts. Despite all these caveats, a recent large-scale analysis of proteins with known function reveals that shifts in rate are good predictors of differing functional classes [75].

Although the role of heterotachy in evolution remains to be clearly defined, the ability to detect rate variation from sequences alone is a powerful resource provided by comparative genomics. I did not use the results to predict functionally important sites in the viral data sets, but the forward-backward algorithm can compute the most likely unobserved state, diverged or not, of each nucleotide site [76]. In a Bayesian context, the prediction can be integrated against the MCMC-approximated posterior density to improve robustness. Specific predictions can generate hypotheses and focus future biological experiment.

## Methods

### Model

We start with an alignment *X *of *N *sequences of length *L*. Nucleotide *X*_*ij *_∈ {*A*, *C*, *G*, *T*, *U*, –} is the nucleotide at position *j *of sequence *i*, which may be a gap (–). Sites *X*._*j *_are treated as independent for all *j *= 1,...,*L*. The evolutionary rate at site *j *is assumed to be selected at random from a discretized gamma distribution with four equi-probable rates and shape parameter *α *[25]. Site-to-site rate variation is greater for smaller *α*, particularly for *α *< 1. Because the actual evolutionary rate of site *j *is unknown, the likelihood of the site *j *data is integrated against all possible rates, using the discrete gamma approximation and removing explicit dependence on the site-specific rate *r*_*j*_. The site likelihood is



where *τ *is the unrooted topology, *t *= (*t*_1_,...,*t*_2*N*-3_) are the branch lengths, *κ *is the transition/transversion ratio assuming the HKY85 nucleotide substitution model [19], and *P*(*r*_*j*_|*α*) = 0.25 is the probability of drawing rate *r*_*j *_from the discrete gamma distribution. Because sites are assumed independent, the full data likelihood is



The model just described is commonly referred to as the variable rates across sites or RAS model.

I now introduce the possibility of a divergence point (DP) into the model. Place a divergence point (DP) in the phylogenetic tree (*τ*, *t*) (Figure 1), on branch *b *∈ {1,...,2*N *- 3} at relative position *l *∈ [0, 1] with respect to an arbitrary root. A fraction *θ *of sites randomly select a new rate after crossing the divergence point, so rate *r*_*j*1 _applies along all the branches in subtree *τ*_1_, including the stub of the branch dissected by the DP and *r*_*j*2 _applies throughout subtree *τ*_2 _along with the other part of the dissected branch. When there is a divergence point, the full site likelihood is



where *X*_(1)*j *_are the site *j *data for sequences of subtree 1 and *X*_(2)*j *_are the data for the sequences of subtree 2. The first term is the likelihood of the site *j *data if the site diverges across the DP, with data *X*_(1)*j *_produced by an evolutionary process with rate *r*_*j*1 _and data *X*_(2)*j *_produced by a process with rate *r*_*j*2_. The second term is the likelihood of site *j *if there is no divergence across the DP and both data *X*_(1)*j *_and *X*_(2)*j*_, i.e. the full site data *X*._*j *_evolve with common rate *r*_*j*_. Again, whether site *j *diverges across the DP is not known, so the full site likelihood is obtained by integrating against the Bernoulli probability distribution for divergence at the DP. If there is no divergence point in the phylogenetic tree, then *θ *= 0 and the site likelihood is given by equation (1) under the RAS model.

### Prior Distributions

Because the model is implemented in a Bayesian context, a prior distribution must be specified for all model parameters. Table 5 lists all prior distributions. Uninformative or nearly uninformative priors are used for most model parameters. The continuous parameters, transition/transversion ratio *κ *∈ [0, ∞), branch lengths *t*_*κ *_∈ [0, ∞), *κ *= l,...,2*N *- 3, and discrete gamma shape parameter *α *∈ [0, ∞) are assumed uniform over a wide range well beyond biologically relevant limits. The DP location *l *∈ [0, 1] is uniform throughout its range. The discrete parameter *b*, indicating the branch of the DP, is uniform over all possible branches. We assume the topology *τ *is known and do not estimate it using the model.

In addition, we place a prior on the presence or absence of a divergence point somewhere in the phylogenetic tree. Let *d *be the number of divergence points in the tree. With probability *P*(*d *= 1) = 0.5 there is a single divergence point at location (*b, l*). Otherwise and also with probability *P*(*d *= 0) = 0.5 there is no divergence point and the RAS model applies. In fact, it may be biologically possible for more than one divergence point to coexist in a phylogenetic tree. We do not consider these more complex models by setting *P*(*d *> 1) = 0.

### MCMC

The posterior distribution is estimated via MCMC using Metropolis-Hastings (MH) within Gibbs sampling. Supplemented with *d*, the parameter vector is now either (1, *κ*, *α*, *t*, *θ*, *l*, *b*) when there is a divergence point or (0, *κ*, *α*, *t*) in the absence of a divergence point. Clearly, the dimension of the parameter space changes with *d *and there are two types of MCMC updates, those within a fixed dimension and trans-dimensional moves. For fixed dimensional updates, the following sequence of moves is applied within a Gibbs cycle to update from (*κ*_*n*_, *α*_*n*_, *t*_*n*_, *θ*_*n*_, *l*_*n*_, *b*_*n*_) to (*κ*_*n*+1_*, α*_*n*+1_, *t*_*n*+1_, *θ*_*n*+1_, *l*_*n*+1_, *b*_*n*+1_) via incremental proposals of (κ*, α*, t*, θ*, l*, b*).

κ* | *κ*_*n*_, *α*_*n*_, *t*_*n*_, *θ*_*n*_, *l*_*n*_, *b*_*n *_= *κ*_*n*_*e*^(*U*-0.5)^, *U *~Unif(0,1)

α* | *κ*_*n*+1_, *α*_*n*_, *t*_*n*_, *θ*_*n*_, *l*_*n*_, *b*_*n *_= Normal(*α*_*n*_, 0.5)

| *κ*_*n*+1_, *α*_*n*+1_, *t*_*ni*_, *θ*_*n*_, *l*_*n*_, *b*_*n *_= *t*_*ni*_*e*^(*U*-0.5)^, U ~Unif(0, 1), *i *= 1,...,2N – 3

θ* | *κ*_*n*+1_, *α*_*n*+1_, *t*_*n*+1_, *θ*_*n*_, *l*_*n*_, *b*_*n *_~ Normal(*θ*_*n*_, 0.2)

l* | *κ*_*n*+1_, *α*_*n*+1_, *t*_*n*+1_, *θ*_*n*+1_, *l*_*n*_, *b*_*n *_~ Normal(*l*_*n*_, 0.2)

b*, l* | κ_*n*+1_, *a*_*n*+1_, *t*_*n*+1_, *θ*_*n*+1_, *l*_*n*_, *b*_*n *_via b* ~ Uniform (1,...,*b *- 1, *b *+ 1,...,2*N *- 3),l* ~ Uniform(0,1) independently.

Here *t*_*ni *_is the *i*th branch length listed in vector *t*_*n *_of the *n*th MCMC sample. Following Minin et al. [68], most parameters defined on the positive real line are updated using an exponential updater that proposes large changes when the parameter is large and small changes when the parameter is small. The DP location, *θ*, and *α *are updated using a reflected normal updater. A large variance is applied because posterior variances tend to be large. To update the branch *b *containing the DP, a joint move is used that proposes a new branch b* uniformly from among all but the current DP branch and proposes a new l* uniformly from the prior Unif(0,1). During each cycle, only one of the last two moves, either updating *l *separately or (*b, l*) jointly, is attempted according to a user-specified mixing probability. For all MCMC samples, this mixing parameter was set to 0.5.

I use reversible jump MCMC [77] to carry out trans-dimensional moves. When proposing to increase *d *from zero to one, the parameter space of the model is supplemented by drawing random variables *z_b_*, *z_l_*, and *z*_*θ *_independently from the prior distributions of *b, l*, and *θ*, respectively. The one-to-one transformation across dimensions is

d* = 1 - *d *

θ* = *z*_*θ*_

κ* = *κ*

l* = *z*_*l*_

α* = *α*

b* = *z*_*b*_

 = *t*_*i *_for all *i*,

which has a Jacobian determinant of one. A trans-dimensional move is attempted during every Gibbs cycle.

Most proposal distributions can be tuned with user-defined tuning parameters. Tuning parameter values used to compute all results in this report are listed in Table 4 along with a summary of all proposal distributions, including Metropolis-Hastings (MH) acceptance ratios. All Metropolis-Hastings acceptance ratios reduce to simple expressions of the likelihoods under the current and proposed states.

The specified model and MCMC algorithm is implemented in a computer program written in C and available upon request from the author.

### Hypothesis Testing

To estimate error rates and power, I compute the Bayes factor [78] in favor of the hypothesis of a divergence point somewhere in the phylogeny vs. the hypothesis of the RAS model without a divergence point. Due to the chosen prior on the divergence point indicator *d*, this Bayes factor is particularly simple



Following [78], log_10 _*B*_*DP *_> 1 is taken to indicate strong support for a divergence point. Conversely, log_10 _*B*_*DP *_< -1 lends strong support to the *absence *of a divergence point. For Bayes factor falling in the region of ambiguity between -1 and 1, no decision can be made with confidence.

It is also possible to compute Bayes factors to test the presence of divergence points on particular branches. Specifically, the Bayes factor in favor of a divergence point along branch *j *is



where *b *is the branch with the divergence point and *P*(*b *= *j*) is the prior probability of a divergence point along branch *j *in the simulation topology. Because each branch is equally likely to carry the divergence point *a priori, P*(*b *= *j*) =  for all *j*. I compute *B*_*j *_when *B*_*DP *_> 1 or regardless of *B*_*DP *_in the case of the HBV data. For the viral data sets I denote *B*_*BC *_and *B*_*FH *_to be the Bayes factors for the branches separating particular named subtrees.

### Simulation

I design a simulation study to verify the code and examine the sensitivity of the method. All sequences are evolved assuming the divergence model described above and assuming the HKY85 [19] model of evolution. I vary *α *∈ {0.01, 0.1, 0.5, 1.0, 2.0} and *θ *∈ {0.0, 0.1, 0.3, 0.5, 0.7, 0.9} to produce a grid of simulation conditions. Note, that the condition *θ *= 0 implies no divergence point. I then simulate 100 datasets for each combination of (*α*, *θ*) assuming the topology of Figure 1, with a DP located at position *l *= 0.9 on the middle branch. All simulated data sets are 1,000 nucleotides long and all branch lengths are fixed at 0.1. At the root, each simulated site is assigned a rate, from a choice of 4 possibilities obtained via equiprobable discretization of the gamma distribution [25]. At the DP, a site is induced to select a new rate class with probability *θ*. Sites that experience a selection shift, i.e. they select a new rate class at the DP, may choose the same rate class with probability 0.25.

I also carry out a number of other simulations. I start by simulating data sets while simultaneously varying *L *∈ {1000, 2500, 5000, 7500}, *t*_*j*_*∈ *{0.03, 0.05, 0.07, 0.09}, *α *∈ {0.7,0.5,0.3,0.1}, and *θ *∈ {0.1, 0.3, 0.5, 0.7}. Each simulation condition is replicated only 10 times and are not shown. They are used principally to select conditions for other more extensive simulations. For example for Figure 4, I simulate 100 data sets of varying length *L *= 1000, 2500, 5000, or 7500 and with varying branch length *t*_*j *_= 0.03, 0.05, 0.07, or 0.09 for all branches *j*. The other parameters are fixed at *α *= 0.7, *θ *= 0.5, and *l *= 0.9. For Figure 5, I simulate 40 data sets either with the divergence point on a terminal branch or on the middle branch, but with varying numbers of taxa, either 2, 4, 6, or 8, in each subtree. When the divergence point is on a terminal branch, the other parameters are *α *= *θ *= 0.5, all branch lengths *t*_*j *_= 0.1, and alignment length is *L *= 1000. When varying the number of taxa, the other parameters are *α *= *θ *= *t*_*j *_= 0.1 and the alignment length *L *= 1000.

Each simulated data set is examined in a single MCMC run of length 6000, burnin 1000, and subsample rate 5. To assess whether this MCMC length, burnin, and subsample are sufficient for convergence, I randomly select one simulated data set per simulation condition and compute a second MCMC sample starting from a distinct initial state. For each pair of MCMC samples, I compute the potential scale reduction factor (PSRF) [54] for the log likelihood and parameters *α*, *κ*, and all branch lengths *t*_*j*_, as well as *θ *and *l *wherever the latter are applicable. The PSRF statistic compares the between sample variance to within sample variance and should be near 1. Of 150 PSRF statistics computed, 2 are above 1.1 and 15 are above 1.01. In addition, I perform a test of proportions for the posterior support of a divergence point, and found 2 significant results at the level of 0.05, for a error rate of about 0.07. In neither case, did the classification of the sample as supporting heterotachy or homotachy differ between the samples.

### Comparison to existing methods

To compare the proposed method with existing methods for detecting temporal rate variation at specific branches, I implement two techniques and apply them to the simulated data. Both techniques rely on specifying two groups of sequences *a priori*. Naturally, the groups I utilize are 0, 1, 2, 3 from subtree 1 and 4, 5, 6, 7 from subtree 2 in Figure 1.

Ané et al. [33] propose to compare two groups by comparing the proportions of invariable sites. Their test statistic is



where *L*_12 _is the number of sites that vary in both subtrees, *L*_1 _is the number of sites that vary in subtree 1, and *L*_2 _is the number of sites that vary in subtree 2. When the two subtrees are completely independent, *W *= 0, however because of a shared ancestor nucleotide and site-to-site rate variation, *W *will usually exceed zero. The presence of a divergence point on the branch separating the two subtrees will decrease *W *by increasing the independence of rates between the two subtrees. Parametric bootstrapping is used to determine whether *W *is significantly smaller than would be expected given statistical variation under the RAS model. For each simulated data set, I estimate *α*, *κ*, stationary nucleotide frequencies *π *and branch lengths *t *using PHYML [79]. I do not estimate, rather assume the true topology. Seq-Gen [80] generates 100 parametric bootstrap datasets under the RAS model using the PHYML-generated parameter estimates, and the *W *statistic is computed for each. The proportion of bootstrap replicates whose statistic falls below the *W *observed for the original simulated dataset is the p-value for rejecting the RAS model.

Lopez et al. [13] describe another test for comparing not just the invariant sites, but the distribution of mutations at all variable sites between two subtrees. One first estimates the number of mutations in each subtree, using the method of Gu and Zhang [81]. Because the Gu and Zhang method returns non-integer estimates of the number of mutations within the subtrees, I round these numbers to the nearest integer before preceeding. The distribution of mutations across sites is then compared between the two subtrees using a chi-square statistic for a 2 × *L *table. Since the asymptotic properties of the chi-square distribution generally do not apply to such data, significance is assessed by 100 permutations of the data while keeping the total number of mutations at each site and within each group (i.e. row and column totals) constant.

### Viral data sets

I collect 5 subtype B [GenBank:AB097870, AY037269, AY037270, AY173959, AY180905] and 5 subtype C [GenBank:AF286224, AF457054, AF361874, AF443088, AY463228] sequences from the HIV database [82] and align them using clustalW [83]. The final alignment is 6610 base pairs long and represents 68% of the entire HIV genome. I collect 7 subtype F [GenBank:AB036905, AB036910, AB064316, AF223965, AY090456, AY090461, X69798] and 3 subtype H [GenBank:AB059661, AY090457, AY090460] sequences from GenBank and align them using clustalW [83]. The final alignment is 3215 base pairs long and represents the entire HBV genome. For each viral data set, I produce 6 MCMC samples of size 1000 from a run of length 6000, burnin 1000, and subsample rate 5. To assess convergence, I compute PSRF [54] of all parameters *θ*, *α*, *κ*, *b*, *t*, *l*.

## Figures and Tables

**Table 1:** Estimation of *α*, *θ*, and *l*.

	Estimation of *α*					Estimation of *θ*					Estimation of *l*				
*θ\α*	2.0	1.0	0.5	0.1	0.01	2.0	1.0	0.5	0.1	0.01	2.0	1.0	0.5	0.1	0.01
0.0	2.05	0.99	0.49	0.10	0.04	NA	NA	NA	NA	NA	NA	NA	NA	NA	NA
	1.90	0.61	0.23	0.05	0.07	NA	NA	NA	NA	NA	NA	NA	NA	NA	NA
0.1	2.18	1.07	0.54	0.11	0.05	0.76	0.68	NA	0.17	0.15	0.55	0.58	NA	0.51	0.51
	2.14	0.69	0.28	0.08	0.08	0.99	0.96	NA	0.21	0.17	0.97	0.99	NA	0.97	0.97
0.3	2.77	1.23	0.59	0.11	0.05	NA	0.49	0.38	0.26	0.26	NA	0.53	0.57	0.58	0.57
	3.42	0.91	0.36	0.08	0.08	NA	0.55	0.48	0.22	0.19	NA	0.97	0.97	0.96	0.97
0.5	3.27	1.41	0.60	0.11	0.06	0.79	0.56	0.43	0.40	0.40	0.37	0.56	0.57	0.63	0.61
	4.59	1.21	0.40	0.09	0.10	0.85	0.73	0.47	0.23	0.21	0.88	0.99	0.96	0.96	0.95
0.7	4.87	1.45	0.62	0.12	0.08	0.80	0.62	0.52	0.55	0.54	0.50	0.53	0.58	0.66	0.65
	7.85	1.49	0.40	0.08	0.10	0.89	0.74	0.50	0.24	0.22	0.95	0.96	0.95	0.94	0.94
0.9	6.74	1.62	0.65	0.13	0.10	0.86	0.74	0.66	0.69	0.69	0.47	0.56	0.61	0.67	0.66
	11.44	1.83	0.40	0.08	0.10	0.86	0.72	0.48	0.24	0.23	0.92	0.97	0.94	0.94	0.93

Each pair of entries summarizes the posterior estimation of model parameters *α*, *θ*, and *l *from 100 random data sets simulated assuming various choices of *θ *(rows) and *α *(columns). The first row, where *θ *= 0 is for data simulated *without *a divergence point. First in each pair is the average posterior mean, summarizing accuracy; second is the average width of the 95% Bayesian credible interval, summarizing precision. Statistics for *θ *and *l *are based only on those simulations strongly supporting a divergence point, which may be substantially fewer than 100 simulations. NA means a divergence point was never strongly supported for that simulation condition.

**Table 2:** HIV estimated parameters.

Parameter	Posterior Mean	LBCI	UBCI	PSRF
*θ*	0.27	0.18	0.38	1.01
*l*	0.56	0.21	0.89	1.03
*α*	0.23	0.19	0.26	1.00
*κ*	5.32	4.89	5.77	1.01
*t*_3_	0.01	0.00	0.01	1.02

log_10 _*B*_*DP *_≈ inf (decisive support for)
log_10 _*B*_*BC *_= 4.08 (decisive support for)

The posterior mean, upper and lower 95% Bayesian credible interval bounds (UBCI and LBCI) and potential scale reduction factor (PSRF) [54] for all continuous parameters of the model. Data for the branch length with largest PSRF is reported. The last two rows report the Bayes factor in support of a divergence point and the Bayes factor in support of a divergence point along the middle branch.

**Table 3:** HBV estimated parameters.

Parameter	Posterior Mean	LBCI	UBCI	PSRF
*θ*	0.37	0.01	0.94	1.01
*l*	0.42	0.02	0.96	1.01
*α*	0.04	0.00	0.08	1.03
*κ*	4.00	3.38	4.70	1.00
*t*_11_	0.01	0.00	0.01	1.02

log_10 _*B*_*DP *_= -0.64 (substantial support against)
log_10 _*B*_*FH *_= -0.71 (substantial support against)

See the caption of Table 2. The second Bayes factor is the support for a divergence point along the middle branch separating the two genotypes.

**Table 4:** Proposal distributions.

Param.	Proposal Distribution	Tuning Param.	MH Ratio
*κ*	κ* = *κ *exp [*κ*_*t*_(*U *- 0.5)], *U *~ Unif(0, 1)	*κ*_*t *_= 1.0	
*α*	α* ~ Normal(*α*, *α*_*t*_)	*α*_*t *_= 0.5	
*t*_*i*_	= *t*_*i *_exp [*t*_*t*_(*U *- 0.5)], *U *~ Unif(0, 1)	*t*_*t *_= 1.0	
*θ*	θ* ~ Normal(*θ*, *θ*_*t*_)	*θ*_*t *_= 0.2	
	l* ~ Normal(*l, l*_*t*_)	*l*_*t *_= 0.2	
	b* ~ Unif(1,...,2*N *- 3), l* ~ Unif(0, 1)	NA	
*d*	d* = 1 - *d*	NA	

For each parameter listed, a new value (starred, e.g. κ*) is proposed according to the listed distribution. Branch lengths *t*_*i*_ are updated one at a time for *i *= 1,...,2*N *- 3. Tuning parameters are subscripted by *t*. Updates of either *l *or (*b, l*) are mixed with probability *m*_*t*_. Update of *d *is a trans-dimensional move. The Metropolis-Hastings acceptance ratios are given in the last column. Dependence on parameters not involved in the update is not shown.

**Table 5:** Prior distributions

Parameter	Distribution
*κ*	Unif(0, 1000)
*α*	Unif(0, 100)
*t*_*i*_	Unif(0, 100), for *i = *1,...,2*N *- 3
*θ*	Unif(0, 1)
*l*	Unif(0, 1)
*b*	Unif(l,...,2*N *- 3)
*τ*	Not estimated
*d*	*P*(*d *= 0) = *P*(*d *= 1) = 0.5
